# Trajectory Analysis for Identifying Classes of Attention Deficit Hyperactivity Disorder (ADHD) in Children of the United States

**DOI:** 10.2174/0117450179298863240516070510

**Published:** 2024-05-21

**Authors:** Yu-Sheng Lee, Matthew Evan Sprong, Junu Shrestha, Matthew P. Smeltzer, Heaven Hollender

**Affiliations:** 1 School of Integrated Sciences, Sustainability, and Public Health, College of Health, Science, and Technology, University of Illinois at Springfield, Illinois, United States; 2 School of Public Management and Policy, College of Public Affairs and Education, University of Illinois at Springfield, llinois, United States; 3 Division of Epidemiology, Biostatistics, and Environmental Health, School of Public Health, University of Memphis, Tennessee, United States; 4 School of Health and Human Sciences, Indiana University Indianapolis, Indiana, United States

**Keywords:** Attention deficit hyperactivity disorder, Neurodevelopmental disorder, Preschool, Children, Families, Hyperactivity, Inattention, Impulsivity

## Abstract

**Background:**

Attention Deficit Hyperactivity Disorder (ADHD) is a mental health disorder that affects attention and behavior. People with ADHD frequently encounter challenges in social interactions, facing issues, like social rejection and difficulties in interpersonal relationships, due to their inattention, impulsivity, and hyperactivity.

**Methods:**

A National Longitudinal Survey of Youth (NLSY) database was employed to identify patterns of ADHD symptoms. The children who were born to women in the NLSY study between 1986 and 2014 were included. A total of 1,847 children in the NLSY 1979 cohort whose hyperactivity/inattention score was calculated when they were four years old were eligible for this study. A trajectory modeling method was used to evaluate the trajectory classes. Sex, baseline antisocial score, baseline anxiety score, and baseline depression score were adjusted to build the trajectory model. We used stepwise multivariate logistic regression models to select the risk factors for the identified trajectories.

**Results:**

The trajectory analysis identified six classes for ADHD, including (1) no sign class, (2) few signs since preschool being persistent class, (3) few signs in preschool but no signs later class, (4) few signs in preschool that magnified in elementary school class, (5) few signs in preschool that diminished later class, and (6) many signs since preschool being persistent class. The sensitivity analysis resulted in a similar trajectory pattern, except for the few signs since preschool that magnified later class. Children’s race, breastfeeding status, headstrong score, immature dependent score, peer conflict score, educational level of the mother, baseline antisocial score, baseline anxious/depressed score, and smoking status 12 months prior to the birth of the child were found to be risk factors in the ADHD trajectory classes.

**Conclusion:**

The trajectory classes findings obtained in the current study can (a) assist a researcher in evaluating an intervention (or combination of interventions) that best decreases the long-term impact of ADHD symptoms and (b) allow clinicians to better assess as to which class a child with ADHD belongs so that appropriate intervention can be employed.

## 1 INTRODUCTION

People with Attention Deficit Hyperactivity Disorder (ADHD) frequently encounter challenges in social interactions, facing issues, like social rejection and difficulties in interpersonal relationships, due to their inattention, impulsivity, and hyperactivity [1-9]. These adverse social consequences lead to children’s emotional distress and anguish as well as parental stress levels [7]. The prevalence of ADHD in children aged 2 to 17 has been reported to vary from 2-13%, with specific rates for different age groups, described as follows: 2-5 years (2%), 6-11 years (10%), and 12-17 years (13%) [10-15]. Treatment and healthcare costs for ADHD can be an additional $15,000 annually compared to children without the disorder [16-18].

### 1.1 Assessment and Treatment of ADHD

The American Psychiatric Association (APA) classifies ADHD into three main types: predominantly inattentive presentation, predominantly hyperactive/impulsive presentation, or combined presentation [19]. Diagnosis relies on observations, assignments, and the Diagnostic and Statistical Manual of Mental Disorders, 5th Edition (DSM-5-TR) criteria [20]. Despite lacking biomarkers or conclusive neuroimaging differences, ADHD is recognized as a neurodevelopmental disorder in the DSM-5-TR [19]. Diagnosis involves initial screening, comprehensive assessment (evaluating symptoms' specifics, duration, and impact on functioning), and bio-psychosocial evaluation [21]. Multimodal treatments, including parent training, medication management, counseling, and educational support, are commonly used [22-27]. Tailoring interventions to individual needs improves effectiveness [1, 28], although prevention strategies can also mitigate ADHD risk [29].

### 1.2 Mental Health and Medical-related Issues

As one of the most common neurobehavioral disorders, ADHD is often associated with disruptive, mood, anxiety, and substance abuse [30-32]. Studies across various disciplines and healthcare sectors have demonstrated that the interplay between these health interactions can exacerbate symptoms, complicate treatment regimens, and diminish overall health outcomes [33-38]. While 5% of children have issues with overactivity, inattention, and impulsivity [39], an estimated 80% of children diagnosed with ADHD also have at least one other psychiatric disorder during their lifetime. Physical consequences, such as cardiovascular disease, are potential long-term consequences of continued pharmacological interventions used to treat ADHD [40-43]. Moreover, the DSM IV-TR did not allow for the dual diagnosis of Autism Spectrum Disorder (ASD) and ADHD, whereas the DSM 5 and DSM 5-TR have allowed for the inclusion of these co-occurring diagnoses [44]. In fact, some studies estimate that the comorbidity rate of ADHD with learning disorders (17%) and ASD (49%) may be due to an overlap in symptoms [45, 46].

### 1.3 Machine Learning and ADHD

Machine learning analyses have been conducted on pre-existing data for children with ADHD in an attempt to determine better strategies for assessing the severity of symptoms [47]. These studies have investigated the use of Magnetic Resonance Imaging (MRI) [47] and Positron Emission Tomography (PET) imaging in machine learning to identify clusters of symptoms [48], but this process has yet to become clinically meaningful [49]. The use of machine learning classifiers for differentiating between multiple psychiatric conditions is based on clinical records among the ADHD population, and there is some benefit for healthcare professionals in categorizing symptoms across different domains, rather than specific domains [50].

Analyzing trajectories in ADHD research is complex and offers valuable insights into predictors, the developmental path over time, variations in symptoms, prognosis, and treatment responses. Research has shown children with ADHD who do not receive treatment to have poorer long-term outcomes in 9 major categories (*i.e*., academic, antisocial behavior, driving, non-medicinal drug use/addictive behavior, obesity, occupation, service use, self-esteem, and social function) compared to those who have received treatment, although outcomes have not been reported to improve to normal levels [38]. Extending this prior research to further cluster symptoms within categories of severity (and observe over a length of time) can (1) aid healthcare professionals in educating families on prevention, (2) enable researchers to design tailored interventions based on specific classes instead of a generic approach, and (3) guide the selection of evidence-based treatments tailored to individual classes once interventions are developed and studied. Hence, this study aimed to utilize the National Longitudinal Survey of Youth (NLSY) database to identify patterns of ADHD symptoms. This identification will help inform diverse prevention strategies in clinical and public health settings. Specifically, we aimed to explore the existence of distinct classes of ADHD symptoms and the variables associated with these classes. While prior research has created clusters of symptom groupings for ADHD, we are exploring how hyperactivity/inattention symptoms change (or do not change) over a length of time.

## 2 MATERIALS AND METHODS

### 2.1 Source of Data

The NLSY is a set of surveys that collect multiple information points on the labor market activities and other significant life events of various groups of men and women. The first cohort (NLSY79), sponsored by the United States (US) Bureau of Labor Statistics, included 12,686 persons aged 14 to 22, with the survey beginning in 1979. In this present study, we used the NLSY79 Child and Young Adult cohort (NLSY79CYA), funded by the Eunice Kennedy Shriver National Institute of Child Health and Human Development, which follows the children who were born to the NLSY79 women cohort between 1986 and 2018. To date, 11,545 children have been followed up and interviewed up to 17 rounds in that period (National Longitudinal Surveys, https://nlsinfo.org/content/cohorts/ nlsy79-children). The NLSY and its associated databases have been proven essential in various research fields, including public health [51]. In addition, this longitudinal survey has high retention rates due to the careful design, making it suitable for life course research [51, 52].

### 2.2 Participants

The NLSY79CYA is a public database that does not contain any personal identifiers. After approval by the institutional review board of the primary author’s university (University of Illinois at Springfield; IRB approval number 24-003), 1,847 children born between 1986 and 2014 in the NLSY79CYA cohort whose hyperactivity/inattention score was calculated when they started this study at the age of four were included in the analysis. Two children born in 2014 were the last cohort included in the analysis. Their hyperactivity/inattention scores were recorded in 2018 when they were four years old.

### 2.3 ADHD and Other Predictive Variables

The NLSY79CYA collected information on children’s sex, race, prenatal care (*e.g*., mother had taken vitamins during pregnancy, mother drank alcohol/smoked during 12 months before the birth of the child), whether premature birth or not, low birth weight (5.5 pounds or less), and postnatal care (*e.g*., breastfeed). The Behavior Problems Index (BPI), developed by Nicholas Zill and James Peterson, was used to evaluate children's behavioral problems. It measures the frequency, range, and types of behavior problems in children aged four and above [53]. The BPI consists of items taken from the Achenbach Behavior Problems Checklist [54] and other child behavior scales [55-57]. The BPI is a tool used to evaluate children's behavior based on six domains: anxious/depressed, headstrong, hyperactive, immature dependency, anti-social, and peer conflict/social withdrawal. Mothers report measurements using 3-level items [58]. The hyperactivity score was first obtained when a child was four years old. The scores were then repeatedly measured in the subsequent five rounds of surveys at ages 6, 8, 10, 12, and 14. Based on the BPI, the NLSY79CYA defines the strength of the hyperactivity/inattention score (ADHD signs) using the following five signs (0-5 points): The child 1) has difficulty concentrating/paying attention; 2) is easily confused and seems in a fog; 3) is impulsive or acts without thinking; 4) has trouble getting his/her mind off certain thoughts; 5) is restless, overly active, and cannot sit still.

A child is defined by the antisocial score (0-6 points) as 1) cheats or tells lies; 2) bullies or is cruel/mean to others; 3) does not seem to feel sorry after misbehaving; 4) breaks things deliberately; 5) is disobedient at school; and 6) has trouble getting along with teachers. Anxious/depressed (0-5 points) is defined as follows: 1) has sudden changes in mood or feeling; 2) feels/complains no one loves him/her; 3) is too fearful or anxious; 4) feels worthless or inferior; and 5) is unhappy, sad, or depressed. A headstrong child (0-5 points) refers to he/she 1) is rather high-strung, tense, and nervous; 2) argues too much; 3) is disobedient at home; 4) is stubborn, sullen, or irritable; and 5) has a strong temper and loses it easily. Dependent (0-4 points) means that the child 1) clings to adults; 2) cries too much; 3) demands a lot of attention; and 4) is too dependent on others. Peer problems score (0-3 points) was measured by asking if the child 1) has trouble getting along with other children; 2) is not liked by other children; and 3) is withdrawn and does not get involved with others. The above BPI scores were measured along with the ADHD signs at 4, 6, 8, 10, 12, and 14 years of age.

### 2.4 Statistical Analyses

A trajectory modeling method, a latent class modeling approach, was used to evaluate one or more outcomes over age or time, as in a repeat measurement from a longitudinal study design [59, 60]. One of the members of the trajectory modeling technique family is Group-based Trajectory Modelling (GBTM) [60-62], which is a semi-parametric finite mixture model designed to identify clusters of individuals following a similar progression of some behavior over time [60, 63, 64]. The GBTM assumes that the population comprises a discrete number of distinct groups that can distinguish subgroups/classes of homogeneous individuals by their behavior profiles [63, 64]. It can help identify the uncertainty of latent group membership based on multiple risk factors that influence group membership decision-making [65-69].

### 2.5 Trajectory Model Building

The first step of building the GBTM is determining the number of and the polynomial order of trajectory classes in a population that best fits the data [63, 70]. A SAS PROC TRAJ procedure was used to build the models. We started from a one-class model with a quartic degree polynomial because the PROC TRAJ procedure does not allow a polynomial order greater than four (quartic). We then increased the number of classes with quartic degree polynomials until the model fit the data according to the Bayesian Information Criterion (BIC) and Bayes factor. When the number of classes was confirmed, the second step was to decrease the polynomial orders of the classes until the highest order polynomial for each class was statistically significant (p < 0.05). We used the logged Bayes factor approximation [2*(BICj-BICi)] proposed by Jeffreys and Kass and Raftery to determine the best-fit model [71]. When the Bayes Factor value exceeds 10, it indicates a strong preference for model j over model i. The value between 6 and 10 suggests a moderate preference for model j over model i. If the value is between 2 and 6, it shows some evidence that model j is favored over model i. However, when the value is less than 2, it suggests no difference between the models; thus, model i should be chosen.

Although ADHD affects both boys and girls, previous studies suggest the disease to be more prevalent in boys than girls [72, 73]. Studies have found ADHD to be associated with antisocial behavior [74, 75]. ADHD and depression can coexist. About one in two adults with ADHD and one in three children with ADHD also have an anxiety disorder [76-80]. Thus, sex, baseline antisocial score, baseline anxiety score, and baseline depression score were adjusted as we built the trajectory model. We also conducted the sensitivity analysis, which only included children who had fully completed the six-round surveys with ten years of follow-up.

### 2.6 Stepwise Logistic Regression

We used stepwise multivariate logistic regression models to select the risk factors for the identified trajectories. A SAS PROC LOGISTIC procedure was performed to determine the most parsimonious model using a significant level of 0.20 set for entry and 0.05 for stay [63, 70, 81, 82]. All analyses were performed using SAS package version 9.4 (SAS Institute Inc, NC).

## 3 RESULTS

Six rounds of surveys were available for the analysis. The baseline/first-round survey comprised 1,847 four-year-old children who enrolled in the NLYS79CYA cohort between 1986 and 2016. The subsequent five rounds repeatedly measured children at 6-, 8-, 10-, 12-, and 14 years of age involving 1,607, 1,515, 1,470, 1,406, and 1,355 children, respectively (Table **1**), accounting for a total of 1,000 children who fully completed the six round surveys with ten years of follow-up.

The first-round survey at four years old involved 917 (49.7%) boys and 930 (50.3%) girls; 52.7% were White, followed by Black children (25.6%) and Hispanic (21.7%). In general, there were more girls (50.3% to 51.6%) than boys (48.4% to 49.2%) in the follow-up rounds. Children who were White constituted the majority of the surveys (51.3% to 52.7%). Furthermore, little more than 50% of the children were breastfed, 21.8% had premature births, and 7.3% were low birth weight babies. The children’s mothers reported that an average of 44.7% drank alcohol and 28.0% smoked during the 12 months before the birth of their child. The mean and Standard Deviation (SD) of baseline antisocial, anxious/depressed, headstrong, dependent, and peer conflicts/withdrawn scores are presented in Table **1**.

### 3.1 Trajectory Classes of ADHD Signs

The trajectory analysis identified six classes (Table **2**). We included race, mother’s education level, premature birth, low birth weight, breastfeeding, mother had taken vitamins during pregnancy, mother drank alcohol/smoked during 12 months before the birth of the child, headstrong score, immature dependency score, and peer conflict/social withdrawal score in the stepwise multivariate logistic regression models. The final model indicated race, mother’s education level, mother smoked during 12 months before the birth of the child, breastfeeding, headstrong score, dependent score, and peer conflict/social withdrawal score as associated with the trajectory classes (Tables **S1**-**S6**). The following key comparisons among the six classes were based on the findings of the descriptive statistics in Table **3** and the multivariate logistic regressions in Tables **S1**-**S6**.

### 3.2 Class C1

C1 class (no sign) involved the most girls with the lowest baseline antisocial, anxious/depressed, headstrong, dependent, and peer conflict scores compared to other classes (Table **3**). This class of children had the highest proportion of breastfeeding (68.6%) (Tables **3** and **S1**). Compared to other classes, their mothers had the highest education levels (13.6 years), the highest vitamin use during pregnancy (98.0%), and the lowest smoking rate 12 months before the birth of the child (17.8%). Children in C1 (8.4%) showed no ADHD signs during the 10-year follow-up.

### 3.3 Classes C2 and C3

**Fig. (1):**
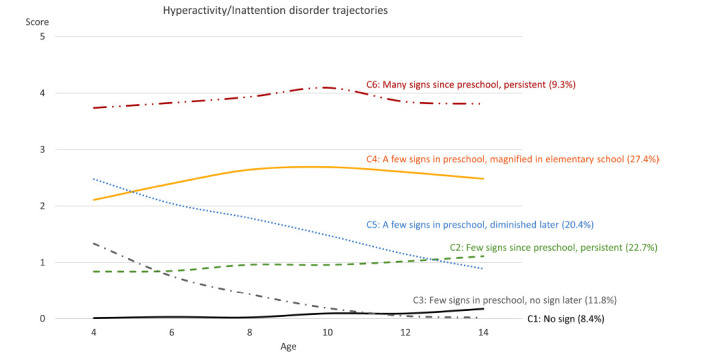
ADHD symptom trajectories for the 6-class model.

**Fig. (2):**
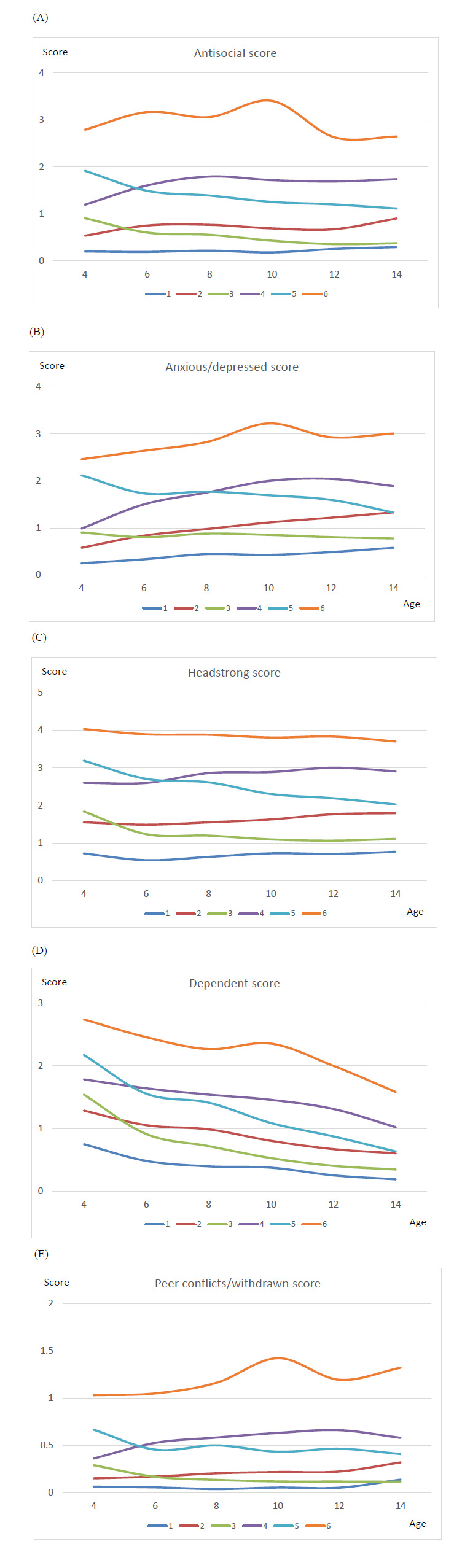
BPI scores at ages 4, 6, 8, 10, 12, and 14 in 6 trajectories.

C2 (few signs since preschool being persistent) and C3 (few signs in preschool but no sign later) classes were similar. C2 children (22.7%) were found to have few signs at their preschool age; the signs persisted throughout the follow-up rounds until the age of 14. These children were considered to be few signs since preschool being persistent class. C3 class (11.8%) included a group of children with few signs at preschool age (Fig. **1**). In contrast to the C2 class, their ADHD signs diminished after they were 12 years old. They were classified as few signs in preschool but no signs later class. Children in both classes had few signs/symptoms since preschool age. However, C3 children had no signs as they grew up, while the signs in the C2 class persisted throughout the surveys. Both classes primarily involved girls (59.8% and 63.0%, respectively) (Table **3**). Compared to the C1 class, children in the C2 and C3 classes had higher BPI scores (Table **S1**). C2 mothers had the highest proportion of alcohol drinking during the 12 months before the birth of the child (48.9%), compared to other classes. In addition, C3 children had the most premature birth (25.5%) compared to other classes. Fig. **2 (A-E)** also demonstrated that among the C3 children, the antisocial, immature dependency, and peer conflict scores decreased in the follow-up rounds. On the other hand, the anxious/depressed score increased in C2 children as they grew up (Fig. **2B**).

### 3.4 Classes C4 and C5

Children in the C4 class (Few signs in preschool that magnified in elementary school) and C5 class (few signs in preschool that diminished later) showed a few signs at baseline (C4 children = 27.4%; C5 children = 20.4%); their (C5) ADHD signs continued diminishing significantly as they grew up to the age of 14. However, for C4 children, the signs were magnified in elementary school (Fig. **1**). The C4 class involved more White boys than C5 (Tables **3** and **S4**). C5 class had more participants within the Black race than C1–C4 (Table **S5**). Although the C5 children had higher BPI scores at age four than the C4 children, the scores tended to decrease afterward. Eventually, the BPI scores in the C5 class were lower than that of the C4 class (Fig. **2**).

### 3.5 Class C6

Children in the C6 class (9.3%) experienced the most signs reported by their mothers. In addition, those signs were persistent throughout the follow-up (Fig. **1**). They were likelier to be Black boys whose mothers had lower education levels (11.9 years) than all other classes (Table **S6**). Also, their mothers had the highest proportion of smoking during the pregnancy (42.5%). Children in this class had the highest BPI scores throughout the follow-up period (Fig. **2**, Tables **3**, and **S6**).

### 3.6 Sensitivity Analysis

**Fig. (3):**
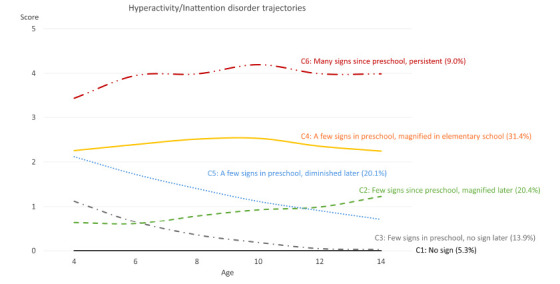
Sensitivity analysis result of ADHD symptom trajectories for the 6-class model.

We included 1,000 out of 1,847 children who fully completed the six-round surveys with ten years of follow-up in the sensitivity analysis. The trajectory analysis also suggested six classes for the ADHD sign patterns (Fig. **3** and Table **S7**). The trajectory pattern of C1 (5.3%), C3 (13.9%), C4 (31.4%), C5 (20.1%), and C6 (9.0%) classes was similar to the whole population. C2 children (20.4%) were found to have few signs at their preschool age; the signs were magnified after they entered elementary school and continued to magnify until age 14. The pattern of this class was slightly different from that of the whole population. These children were defined as a class with a few signs since preschool that magnified later.

## 4 DISCUSSION

ADHD is highly heterogeneous, which has made it extremely challenging for researchers to identify the underlying pathophysiology, developmental trajectories, and effective interventions at the individual level. This study used the NLSY79CYA longitudinal cohort to develop the ADHD trajectories among 1,847 children who were followed up to six rounds between 1986 and 2018. The hyperactivity/inattention scores of two children born in 2014 were obtained in 2018 when they were four years old. Although only one data point was provided in the analyses, the influence on the trajectory patterns could be ignored. In the sensitivity analysis including only children who fully completed the six-round surveys, we did not find significantly different patterns from the whole population. We found the optimal ADHD sign trajectory classes to be no sign class (C1), few signs since preschool being persistent class (C2), few signs in preschool but no signs later class (C3), few signs in preschool that magnified in elementary school class (C4), few signs in preschool that diminished later class (C5), and many signs since preschool being persistent class (C6). The sensitivity analysis resulted in a similar trajectory pattern, except for the few signs since preschool that magnified later class.

The patterns of no sign class (C1) and few signs since preschool being persistent class (C2) were consistent with a previous UK study. However, in our study, 8.4% were in the no sign class, compared to 34.9%, and 22.7% of children were in the few signs since preschool being persistent class compared to 24.1% in the UK population [83]. In another study [84], the authors called the C1 class as “persisting low,” which comprised 38.2% of their study population. The few signs since preschool being persistent class (C2) showed slightly elevated signs compared to the whole population. However, in the sensitivity analysis, we found that this class of children showed significantly magnified signs throughout the follow-up. Compared to children with completed hyperactivity/inattention score records, children who missed at least one round of the survey had lower hyperactivity/inattention scores at ages 10, 12, and 14. These children also had higher antisocial and headstrong scores than the fully surveyed children. Their mothers’ education level was slightly lower than that of the fully surveyed children (13.1 years *vs*. 13.5 years). In the sensitivity analysis, children in the few signs since preschool that magnified later class (C2) had higher baseline peer conflict scores than the whole population.

The findings of a few signs in preschool but no sign later class (C3) were in accordance with Leopold and colleagues’ study findings. Their study found that hyperactivity/impulsivity declined from preschool through ninth grade (14 years old) in a community twin sample [85]. Another study among children from low-income families followed from kindergarten through third grade (8 years old) also demonstrated a similar pattern [86]. Those children had few signs of inattention in kindergarten but they declined later. Many children with higher levels of ADHD signs [*e.g*., a few signs in preschool that magnified in elementary school class (C4) and many signs since preschool being persistent class (C6)] developed ADHD during the school-age years [87]. The findings in this present study of a few signs in preschool that magnified in elementary school class (C4) and many signs since preschool being persistent class (C6) have been found to be consistent with previous studies. A few signs in preschool that magnified in elementary school class (C4) was also called “persisters” in O’Neill’s study. They described that children in this class had high ADHD symptoms level in preschool age and it continued to persist with higher level symptoms throughout the school age. They eventually met the diagnostic criteria for ADHD [87]. The many signs since preschool being persistent class (C6) was the classical ADHD trajectory class found in previous studies [83, 88]. This class of children included less girls and had more behavior/conduct problems than other classes. This finding has been found to be consistent with other studies [83, 89]. A previous study also called it “pre-school onset being persistent.” [83] A study was conducted on the US children recruited from schools located in high-risk communities across four states (NC, TN, WA, and PA) between 3rd and 12th grades. The study found a similar class, but it did not include younger children, unlike the current study [90].

The findings of a few signs in preschool that diminished later class (C5) have also been found to be consistent with previous studies [83, 84, 87]. In O’Neill’s study, this class was defined as “preschool-limited”. Murray and colleagues called it “subclinical remitting.” Tandon and colleagues called it “gradually remitting.” A study investigating the trajectory patterns among US children between 3 and 5 years of age also found that some children demonstrated a similar pattern. Those children were reported with high ADHD symptoms at age 3. Some ADHD symptoms diminished as they grew up to 5 years old [88].

### 4.1 Risk Factors

Understanding the factors that influence trajectories in various phenomena is crucial for informed decision-making and effective interventions. However, identifying the risk factors associated with these trajectories is pivotal, as it not only enhances our comprehension of the underlying processes, but also enables the development of targeted and timely interventions. By uncovering the elements that predispose individuals to specific trajectories, researchers and policymakers gain valuable insights that can inform preventive strategies [91], personalized treatments, and public health initiatives. The current study’s findings showed children’s race, breastfeeding status, headstrong score, immature dependent score, peer conflict score, educational level of the mother, baseline antisocial score, baseline anxious/depressed score, and smoking status 12 months prior to the birth of the child as risk factors in the ADHD trajectory classes (after adjusting for children’s sex, baseline antisocial score, and baseline anxious/depressed score).

### 4.2 Race and Educational Level

Race was a potential risk factor among the trajectory classes. Studies have found the minority children (*e.g*., African Americans or Hispanics) to be much less likely than identical White children to receive an ADHD diagnosis [92, 93]. These racial differences in ADHD diagnosis occur as early as kindergarten and continue until middle school. These children are also less likely to be using medication to treat the disorder by the end of elementary and middle school [92]. Studies have also revealed maternal breastfeeding to be an important factor for a lower risk of ADHD in children [94, 95]. This has been found to be consistent with our findings in this study.

Mother’s education level has also been found to be a predictor for the trajectory classes [83]. Studies have found children with low ADHD symptom classes to have mothers with higher education levels compared to those with high ADHD symptom classes. Prenatal smoking has been found to be a risk factor for ADHD trajectory classes. High ADHD symptoms have been found to be associated with a higher proportion of mothers’ prenatal smoking [88, 96, 97].

The limitations of the current study are as follows: secondary data sources can have limitations in terms of accuracy, completeness, and relevance. The quality of the trajectory analysis heavily depends on the quality of the data source, and inaccurate or incomplete data could lead to biased results or limited generalizability of findings. Additionally, specific variables (*e.g*., ADHD diagnosis – yes/no) that could generate greater discovery may limit the depth of the analysis and the ability to draw meaningful conclusions. Secondary data may have also lacked detailed contextual information related to the individuals in the dataset, which more rigorous non-quasi-experimental analysis or qualitative methodologies may provide greater context as to why the trends we found have existed. Specific to the current study, the hyperactive index only investigated part of the ADHD symptoms. A comprehensive screening and diagnosis by a licensed clinician based on the DSM-5-TR, coupled with the variables identified within the current study, would yield more robust predictions that could result in earlier intervention and determine the best course of intervention. Finally, we have not included the family history of prior ADHD diagnosis in our predictive model (data were unavailable). It is strongly supported that parents’ diagnosis of ADHD strongly predicts whether the child will have a similar diagnosis [98].

## CONCLUSION

Previous studies have demonstrated two key findings: firstly, environmental influences play a significant role in shaping both the structure and function of the developing brain, and secondly, alterations in brain structure and function are directly linked to ADHD as individuals progress through developmental stages [29]. Notably, research has emphasized the critical window of opportunity during the preschool years when the brain exhibits higher plasticity, making it more receptive to lasting modifications, and prior to the emergence of complicating factors that can hinder treatment efficacy.

Interventions implemented during this early stage have been proven to mitigate the long-term impact of ADHD trajectories. Recent studies have underscored the positive outcomes of preschool-age interventions, revealing significant improvements in social skills and reductions in behavioral problems according to assessments from teachers and parents [99, 100]. Additionally, research has highlighted the effectiveness of early intervention programs tailored for young children aged 2 to 5, emphasizing the enhancement of self-regulatory behaviors and fostering stronger interpersonal relationships between educators and parents [101, 102]. Among the consistently successful strategies found in these intervention programs have been parent management techniques and preschool teacher training. These methods encompass diverse learning materials, such as parent-child interaction training, psychoeducation, behavioral interventions within the preschool setting, and child-focused approaches, like social and positive reinforcement for adhering to rules [103].

Using the trajectory classes as identified in the current study can (a) assist a researcher in evaluating an intervention (or combination of interventions) that best decreases the long-term impact of ADHD symptoms and (b) allow clinicians to better assess which class a child with ADHD belongs to so that appropriate intervention can be employed.

## Figures and Tables

**Table 1:** Sample characteristics and ADHD predictors by age.

-	4 Years	6 Years	8 Years	10 Years	12 Years	14 Years
Number of children in the survey	1847	1607	1515	1470	1406	1355
Hyperactivity/inattention score (0-5; mean, SD)	1.79 (1.46)	1.70 (1.53)	1.72 (1.57)	1.65 (1.60)	1.53 (1.55)	1.46 (1.54)
Sex	-	-	-	-	-	-
Boy	917 (49.7%)	781 (48.6%)	733 (48.4%)	715 (48.6%)	682 (48.5%)	667 (49.2%)
Girl	930 (50.3%)	826 (51.4%)	782 (51.6%)	755 (51.4%)	724 (51.5%)	688 (50.3%)
Race	-	-	-	-	-	-
Hispanic	401 (21.7%)	354 (22.0%)	328 (21.7%)	322 (21.9%)	294 (20.9%)	287 (21.3%)
Black	472 (25.6%)	422 (26.3%)	398 (26.3%)	392 (26.7%)	379 (27.0%)	317 (27.4%)
White, non-Hispanic	974 (52.7%)	831 (51.7%)	789 (52.0%)	756 (51.4%)	733 (52.1%)	695 (51.3%)
Premature birth	-	-	-	-	-	-
No	1445 (78.2%)	-	-	-	-	-
Yes	402 (21.8%)	-	-	-	-	-
Low birth weight	-	-	-	-	-	-
No	1537 (92.7%)	-	-	-	-	-
Yes	121 (7.3%)	-	-	-	-	-
Breastfeed	-	-	-	-	-	-
No	858 (48.7%)	-	-	-	-	-
Yes	902 (51.3%)	-	-	-	-	-
Mother’s education level (year, SD)	12.8 (2.46)	-	-	-	-	-
Mother drank alcohol during 12 months before the birth of child (yes)	-	-	-	-	-	-
No	931 (55.3%)	-	-	-	-	-
Yes	752 (44.7%)	-	-	-	-	-
Mother smoked during 12 months before the birth of the child (yes)	-	-	-	-	-	-
No	1203 (72.0%)	-	-	-	-	-
Yes	467 (28.0%)	-	-	-	-	-
Antisocial score (0-6; mean, SD)	1.20 (1.20)	-	-	-	-	-
Anxious/depressed score (0-5; mean, SD)	1.16 (1.17)	-	-	-	-	-
Headstrong score (0-5; mean, SD)	2.36 (1.61)	-	-	-	-	-
Dependent score (0-4; mean, SD)	1.71 (1.29)	-	-	-	-	-
Peer conflicts/withdrawn score (0-3; mean, SD)	0.40 (0.71)	-	-	-	-	-

**Table 2:** Model fit for 1-7 class quartic group-based trajectory analysis.

Model	Number of Class	BIC	Jeffreys and Kass and Raftery Approximation 2*(BICj-BICi)	Model Comparison (j to i)	Evidence for or Against
1	One	-16293.82	--	--	--
2	Two	-14821.21	2945.22	Model 2 to model 1	Very strong evidence against model 1
3	Three	-14532.50	577.42	Model 3 to model 2	Very strong evidence against model 2
4	Four	-14442.39	180.22	Model 4 to model 3	Very strong evidence against model 3
5	Five	-14391.91	100.96	Model 5 to model 4	Very strong evidence against model 4
6	**Six**	-14373.36	**37.10**	Model 6 to model 5	Strong evidence against model 5
7	Seven	-14390.67	-34.62	Model 7 to model 6	No evidence against model 6

**Table 3:** Descriptive statistics for six classes of ADHD.

-	C1 (No Sign)	C2 (Few Signs Since Preschool Being Persistent)	C3 (Few Signs in Preschool but No Sign Later)	C4 (Few Signs in Preschool that Magnified in Elementary School)	C5 (Few Signs in Preschool that Diminished Later)	C6 (Many Signs Since Preschool being Persistent)
% of children	8.4% (n=160)	22.7% (n=418)	11.8% (n=200)	27.4% (n=544)	20.4% (n=341)	9.3% (n=162)
Sex	-	-	-	-	-	-
Boy	58 (36.3%)	168 (40.2%)	74 (37.0%)	338 (62.1%)	158 (46.3%)	111 (68.5%)
Girl	102 (63.7%)	250 (59.8%)	126 (63.0%)	206 (37.9%)	183 (53.7%)	51 (31.5%)
Race	-	-	-	-	-	-
Hispanic	33 (20.6%)	82 (19.6%)	49 (24.5%)	128 (23.5%)	72 (21.1%)	31 (19.1%)
Black	28 (17.5%)	104 (24.9%)	42 (21.0%)	129 (23.7%)	113 (33.1%)	52 (32.1%)
White, non-Hispanic	99 (61.9%)	232 (55.5%)	109 (54.5%)	287 (52.8%)	156 (45.8%)	79 (48.8%)
Premature birth	-	-	-	-	-	-
No	130 (81.3%)	330 (79.0%)	149 (74.5%)	432 (79.4%)	257 (75.4%)	128 (79.0%)
Yes	30 (18.7%)	88 (21.0%)	51 (25.5%)	112 (20.6%)	84 (24.6%)	34 (21.0%)
Low birth weight	-	-	-	-	-	-
No	136 (94.4%)	348 (93.3%)	164 (92.7%)	452 (92.6%)	278 (90.9%)	140 (93.3%)
Yes	8 (5.6%)	25 (6.7%)	13 (7.3%)	36 (7.4%)	28 (9.1%)	10 (6.7%)
Breastfeed	-	-	-	-	-	-
No	49 (31.4%)	182 (46.0%)	91 (47.2%)	271 (52.2%)	164 (51.1%)	91 (58.3%)
Yes	107 (68.6%)	214 (54.0%)	102 (52.8%)	248 (47.8%)	157 (48.9%)	65 (41.7%)
Mother’s education level (year, SD)	13.6 (2.40)	13.3 (2.46)	12.9 (2.52)	12.4 (2.46)	12.6 (2.31)	11.9 (2.16)
Mother took vitamins during pregnancy	-	-	-	-	-	-
No	3 (2.0%)	16 (4.2%)	8 (4.6%)	27 (5.5%)	12 (4.0%)	5 (3.3%)
Yes	144 (98.0%)	361 (95.8%)	166 (95.4)	468 (94.5%)	292 (96.0%)	147 (96.7%)
Mother drank alcohol during 12 months before the birth of child (yes)	-	-	-	-	-	-
No	83 (56.5%)	194 (51.1%)	102 (57.6%)	274 (54.9%)	177 (57.5%)	87 (57.2%)
Yes	64 (43.5%)	186 (48.9%)	75 (42.4%)	225 (45.1%)	131 (42.5%)	65 (42.8%)
Mother smoked during 12 months before the birth of the child (yes)	-	-	-	-	-	-
No	120 (82.2%)	291 (76.8%)	134 (76.1%)	333 (67.3%)	222 (73.3%)	86 (57.0%)
Yes	26 (17.8%)	88 (23.2%)	42 (23.9%)	162 (32.7%)	81 (26.7%)	65 (43.0%)
Baseline antisocial score (0-6; mean, SD)	0.20 (0.45)	0.54 (0.75)	0.91 (1.00)	1.19 (1.03)	1.92 (1.13)	2.79 (1.05)
Baseline anxious/depressed score (0-5; mean, SD)	0.25 (0.50)	0.59 (0.73)	0.91 (0.88)	0.99 (0.87)	2.12 (1.22)	2.46 (1.31)
Baseline headstrong score (0-5; mean, SD)	0.72 (1.05)	1.55 (1.34)	1.84 (1.43)	2.60 (1.45)	3.19 (1.31)	4.03 (1.11)
Baseline dependent score (0-4; mean, SD)	0.75 (1.10)	1.28 (1.16)	1.54 (1.21)	1.78 (1.21)	2.17 (1.18)	2.74 (1.25)
Baseline peer conflicts/withdrawn score (0-3; mean, SD)	0.06 (0.24)	0.15 (0.41)	0.29 (0.59)	0.36 (0.62)	0.67 (0.86)	1.03 (0.99)

## Data Availability

The anonymized NLSY data collected are available as open database at https://nlsinfo.org/content/cohorts/ nlsy79-children.

## LIST OF ABBREVIATIONS

NLSY79: 1979 Cohort of National Longitudinal Survey of Youth
NLSY79CYA: NLSY79 Child and Young Adult
ADHD: Attention Deficit Hyperactivity Disorder
DSM-5-TR: Diagnostic and Statistical Manual of Mental Disorders, 5th Edition
BIC: Bayesian Information Criterion
BPI: Behavior Problems Index
GBTM: Group-based Trajectory Model

## STANDARDS OF REPORTING

STROBE guidelines were followed.
