# Supplementary material for: Trajectory Analysis for Identifying Classes of Attention Deficit Hyperactivity Disorder (ADHD) in Children of the United States
Source: Clin Pract Epidemiol Ment Health. 2024 May 21;20:e17450179298863. doi: 10.2174/0117450179298863240516070510 (PMC11311732; doi:10.2174/0117450179298863240516070510)
Supplement: Supplementary file 1 [file CPEMH-20-E17450179298863_SD1.pdf]

Clin. Pract. Epidemiol. Ment. Health
ISSN: 1745-0179
DOI: 10.2174/0117450179298863240516070510, 2024, 20, e17450179298863

SUPPLEMENTARY MATERIAL
OPEN ACCESS

Trajectory Analysis for Identifying Classes of Attention Deficit Hyperactivity Disorder (ADHD) in Children of the United States

Yu-Sheng Lee[1], Matthew Evan Sprong[2,*], Junu Shrestha[1], Matthew P. Smeltzer[3] and Heaven Hollender[4]

[1]*School of Integrated Sciences, Sustainability, and Public Health, College of Health, Science, and Technology, University of Illinois at Springfield, United States*
[2]*School of Public Management and Policy, College of Public Affairs and Education, University of Illinois at Springfield, United States*
[3]*Division of Epidemiology, Biostatistics, and Environmental Health, School of Public Health, University of Memphis, United States*
[4]*School of Health and Human Sciences, Indiana University-Purdue University Indianapolis, United States*

© 2024 The Author(s). Published by Bentham Open.
This is an open access article distributed under the terms of the Creative Commons Attribution 4.0 International Public License (CC-BY 4.0), a copy of which is available at: https://creativecommons.org/licenses/by/4.0/legalcode. This license permits unrestricted use, distribution, and reproduction in any medium, provided the original author and source are credited.

*Address correspondence to this author at the School of Public Management and Policy, College of Public Affairs and Education, University of Illinois at Springfield, PAC 478, One University Plaza, Springfield, Illinois, 62703, United States; Tel: 217-206-7579; E-mail: mspro2@uis.edu

Published: May 21, 2024

Cite as: Lee Y, Sprong M, Shrestha J, Smeltzer M, Hollender H. Trajectory Analysis for Identifying Classes of Attention Deficit Hyperactivity Disorder (ADHD) in Children of the United States. Clin Pract Epidemiol Ment Health, 2024; 20: e17450179298863. http://dx.doi.org/10.2174/0117450179298863240516070510

Send Orders for Reprints to reprints@benthamscience.net

Table S1. Logistic regression stepwise selected predictors using C1 as the reference group.

-	Odds Ratio	95% CI	p-value
**C2**	-	-	-
Race	-	-	-
Hispanic vs. White, non-Hispanic	1.07	(0.62 - 1.84)	0.6427
Black vs. White, non-Hispanic	1.47	(0.85 - 2.54)	0.2056
Mother’s education level (year)	1.02	(0.93 - 1.12)	0.7305
Mother smoked during 12 months before the birth of the child (yes)	1.20	(0.71 - 2.04)	0.5020
Breastfeed (yes)	0.56	(0.36 - 0.89)	0.0140*
Headstrong score	1.90	(1.55 - 2.33)	<.0001*
Dependent score	1.24	(1.01 - 1.53)	0.0364*
Peer conflict/social withdrawal score	1.57	(0.77 - 3.16)	0.2124
-	-	-	-
**C3**	-	-	-
Race	-	-	-
Hispanic vs. White, non-Hispanic	1.50	(0.82 - 2.77)	0.3317
Black vs. White, non-Hispanic	1.25	(0.66 - 2.35)	0.9626
Mother’s education level (year)	0.96	(0.86 - 1.07)	0.4385
Mother smoked during 12 months before the birth of the child (yes)	1.11	(0.61 - 2.03)	0.7263
Breastfeed (yes)	0.50	(0.30 - 0.85)	0.0094*
Headstrong score	1.97	(1.58 - 2.47)	<.0001*

-	Odds Ratio	95% CI	p-value
Dependent score	1.42	(1.14 - 1.78)	0.0021*
Peer conflict/social withdrawal score	2.57	(1.26 - 5.24)	0.0095*
-	-	-	-
**C4**	-	-	-
Race	-	-	-
Hispanic vs. White, non-Hispanic	1.62	(0.93 - 2.83)	0.4804
Black vs. White, non-Hispanic	1.76	(1.00 - 3.11)	0.2502
Mother's education level (year)	0.91	(0.83 - 1.01)	0.0634
Mother smoked during 12 months before the birth of the child (yes)	1.65	(0.96 - 2.81)	0.0686
Breastfeed (yes)	0.53	(0.33 - 0.85)	0.0086*
Headstrong score	2.88	(2.34 - 3.54)	<.0001*
Dependent score	1.46	(1.18 - 1.79)	0.0004*
Peer conflict/social withdrawal score	2.37	(1.19 - 4.73)	0.0142*
-	-	-	-
**C5**	-	-	-
Race	-	-	-
Hispanic vs. White, non-Hispanic	1.67	(0.89 - 3.11)	0.8532
Black vs. White, non-Hispanic	3.11	(1.69 - 5.72)	0.0037*
Mother's education level (year)	0.95	(0.85 - 1.05)	0.2970
Mother smoked during 12 months before the birth of child (yes)	1.24	(0.69 - 2.24)	0.4661
Breastfeed (yes)	0.59	(0.35 - 0.98)	0.0418*
Headstrong score	3.63	(2.91 - 4.53)	<.0001*
Dependent score	1.63	(1.30 - 2.03)	<.0001*
Peer conflict/social withdrawal score	3.60	(1.79 - 7.22)	0.0003*
-	-	-	-
**C6**	-	-	-
Race	-	-	-
Hispanic vs. White, non-Hispanic	1.64	(0.76 - 3.52)	0.6985
Black vs. White, non-Hispanic	3.59	(1.76 - 7.31)	0.0036*
Mother's education level (year)	0.83	(0.74 - 0.95)	0.0048*
Mother smoked during 12 months before the birth of the child (yes)	2.20	(1.14 - 4.27)	0.0192*
Breastfeed (yes)	0.57	(0.31 - 1.04)	0.0659
Headstrong score	5.65	(4.31 - 7.41)	<.0001*
Dependent score	2.15	(1.65 - 2.80)	<.0001*
Peer conflict/social withdrawal score	4.29	(2.10 - 8.77)	<.0001*

Table S2. Logistic regression stepwise selected predictors using C2 as the reference group.

-	Odds Ratio	95% CI	p-value
**C1**	-	-	-
Race	-	-	-
Hispanic vs. White, non-Hispanic	0.94	(0.54 - 1.62)	0.6427
Black vs. White, non-Hispanic	0.68	(0.39 - 1.18)	0.2056
Mother's education level (year)	0.98	(0.90 - 1.08)	0.7305
Mother smoked during 12 months before the birth of the child (yes)	0.83	(0.49 - 1.42)	0.5020
Breastfeed (yes)	1.78	(1.12 - 2.80)	0.0140*
Headstrong score	0.53	(0.43 - 0.65)	<.0001*
Dependent score	0.80	(0.66 - 0.99)	0.0364*
Peer conflict/social withdrawal score	0.64	(0.32 - 1.29)	0.2124
-	-	-	-
**C3**	-	-	-
Race	-	-	-
Hispanic vs. White, non-Hispanic	1.41	(0.87 - 2.29)	0.0744
Black vs. White, non-Hispanic	0.85	(0.52 - 1.38)	0.1584
Mother's education level (year)	0.94	(0.87 - 1.03)	0.1712

-	Odds Ratio	95% CI	p-value
Mother smoked during 12 months before the birth of the child (yes)	0.93	(0.59 - 1.46)	0.7458
Breastfeed (yes)	0.89	(0.60 - 1.33)	0.5739
Headstrong score	1.04	(0.91 - 1.20)	0.5689
Dependent score	1.15	(0.97 - 1.35)	0.0998
Peer conflict/social withdrawal score	1.64	(1.14 - 2.37)	0.0078*
-	-	-	-
**C4**	-	-	-
Race	-	-	-
Hispanic vs. White, non-Hispanic	1.52	(1.02 - 2.26)	0.0931
Black vs. White, non-Hispanic	1.20	(0.83 - 1.74)	0.8920
Mother's education level (year)	0.90	(0.84 - 0.96)	0.0017*
Mother smoked during 12 months before the birth of the child (yes)	1.37	(0.98 - 1.93)	0.0688
Breastfeed (yes)	0.95	(0.69 - 1.29)	0.7291
Headstrong score	1.52	(1.36 - 1.69)	<.0001*
Dependent score	1.17	(1.03 - 1.33)	0.0161*
Peer conflict/social withdrawal score	1.52	(1.12 - 2.05)	0.0070*
-	-	-	-
**C5**	-	-	-
Race	-	-	-
Hispanic vs. White, non-Hispanic	1.56	(0.97 - 2.53)	0.7588
Black vs. White, non-Hispanic	2.12	(1.39 - 3.24)	0.0113*
Mother's education level (year)	0.93	(0.86 - 1.01)	0.0709
Mother smoked during 12 months before the birth of the child (yes)	1.04	(0.69 - 1.56)	0.8619
Breastfeed (yes)	1.04	(0.72 - 1.50)	0.8257
Headstrong score	1.92	(1.68 - 2.19)	<.0001*
Dependent score	1.31	(1.13 - 1.52)	0.0004*
Peer conflict/social withdrawal score	2.30	(1.68 - 3.14)	<.0001*
-	-	-	-
**C6**	-	-	-
Race	-	-	-
Hispanic vs. White, non-Hispanic	1.54	(0.80 - 2.94)	0.9564
Black vs. White, non-Hispanic	2.45	(1.40 - 4.27)	0.0138*
Mother's education level (year)	0.82	(0.74 - 0.91)	0.0002*
Mother smoked during 12 months before the birth of the child (yes)	1.84	(1.11 - 3.05)	0.0187*
Breastfeed (yes)	1.01	(0.62 - 1.63)	0.9731
Headstrong score	2.98	(2.43 - 3.66)	<.0001*
Dependent score	1.73	(1.41 - 2.12)	<.0001*
Peer conflict/social withdrawal score	2.74	(1.94 - 3.88)	<.0001*

Table S3. Logistic regression stepwise selected predictors using C3 as the reference group.

-	Odds Ratio	95% CI	p-value
**C1**	-	-	-
Race	-	-	-
Hispanic vs. White, non-Hispanic	0.67	(0.36 - 1.23)	0.3317
Black vs. White, non-Hispanic	0.80	(0.43 - 1.52)	0.9626
Mother's education level (year)	1.04	(0.94 - 1.16)	0.4385
Mother smoked during 12 months before the birth of the child (yes)	0.90	(0.49 - 1.64)	0.7263
Breastfeed (yes)	1.99	(1.18 - 3.34)	0.0094*
Headstrong score	0.51	(0.41 - 0.63)	<.0001*
Dependent score	0.70	(0.56 - 0.88)	0.0021*
Peer conflict/social withdrawal score	0.39	(0.19 - 0.79)	0.0095*
-	-	-	-
**C2**	-	-	-
Race	-	-	-

-	Odds Ratio	95% CI	p-value
Hispanic vs. White, non-Hispanic	0.71	(0.44 - 1.15)	0.0744
Black vs. White, non-Hispanic	1.18	(0.73 - 1.91)	0.1584
Mother's education level (year)	1.06	(0.98 - 1.15)	0.1712
Mother smoked during 12 months before the birth of the child (yes)	1.08	(0.69 - 1.69)	0.7458
Breastfeed (yes)	1.12	(0.76 - 1.66)	0.5739
Headstrong score	0.96	(0.83 - 1.11)	0.5689
Dependent score	0.87	(0.74 - 1.03)	0.0998
Peer conflict/social withdrawal score	0.61	(0.42 - 0.88)	0.0078*
-	-	-	-
**C4**	-	-	-
Race	-	-	-
Hispanic vs. White, non-Hispanic	1.08	(0.68 - 1.71)	0.6549
Black vs. White, non-Hispanic	1.42	(0.88 - 2.28)	0.1776
Mother's education level (year)	0.95	(0.88 - 1.03)	0.2291
Mother smoked during 12 months before the birth of the child (yes)	1.48	(0.97 - 2.26)	0.0709
Breastfeed (yes)	1.06	(0.73 - 1.55)	0.7639
Headstrong score	1.46	(1.28 - 1.67)	<.0001*
Dependent score	1.02	(0.88 - 1.19)	0.7887
Peer conflict/social withdrawal score	0.92	(0.68 - 1.25)	0.6014
-	-	-	-
**C5**	-	-	-
Race	-	-	-
Hispanic vs. White, non-Hispanic	1.11	(0.65 - 1.88)	0.1646
Black vs. White, non-Hispanic	2.50	(1.50 - 4.18)	0.0005*
Mother's education level (year)	0.99	(0.90 - 1.08)	0.7555
Mother smoked during 12 months before the birth of the child (yes)	1.12	(0.69 - 1.80)	0.6486
Breastfeed (yes)	1.17	(0.76 - 1.78)	0.4746
Headstrong score	1.84	(1.58 - 2.14)	<.0001*
Dependent score	1.14	(0.96 - 1.36)	0.1245
Peer conflict/social withdrawal score	1.40	(1.03 - 1.90)	0.0322*
-	-	-	-
**C6**	-	-	-
Race	-	-	-
Hispanic vs. White, non-Hispanic	1.09	(0.55 - 2.16)	0.1782
Black vs. White, non-Hispanic	2.88	(1.54 - 5.39)	0.0009*
Mother's education level (year)	0.87	(0.78 - 0.97)	0.0146*
Mother smoked during 12 months before the birth of the child (yes)	1.98	(1.13 - 3.47)	0.0170*
Breastfeed (yes)	1.13	(0.67 - 1.91)	0.6497
Headstrong score	2.86	(2.31 - 3.55)	<.0001*
Dependent score	1.51	(1.21 - 1.88)	0.0003*
Peer conflict/social withdrawal score	1.67	(1.19 - 2.35)	0.0032*

Table S4. Logistic regression stepwise selected predictors using C4 as the reference group.

-	Odds Ratio	95% CI	p-value
**C1**	-	-	-
Race	-	-	-
Hispanic vs. White, non-Hispanic	0.62	(0.35 - 1.08)	0.4804
Black vs. White, non-Hispanic	0.57	(0.32 - 1.00)	0.2502
Mother's education level (year)	1.10	(1.00 - 1.21)	0.0634
Mother smoked during 12 months before the birth of the child (yes)	0.61	(0.36 - 1.04)	0.0686
Breastfeed (yes)	1.88	(1.17 - 3.00)	0.0086*
Headstrong score	0.35	(0.28 - 0.43)	<.0001*
Dependent score	0.69	(0.56 - 0.85)	0.0004*
Peer conflict/social withdrawal score	0.42	(0.21 - 0.84)	0.0142*

-	Odds Ratio	95% CI	p-value
-	-	-	-
**C2**	-	-	-
Race	-	-	-
Hispanic vs. White, non-Hispanic	0.66	(0.44 - 0.98)	0.0931
Black vs. White, non-Hispanic	0.83	(0.58 - 1.20)	0.8920
Mother’s education level (year)	1.12	(1.04 - 1.19)	0.0017*
Mother smoked during 12 months before the birth of the child (yes)	0.73	(0.52 - 1.03)	0.0688
Breastfeed (yes)	1.06	(0.77 - 1.44)	0.7291
Headstrong score	0.66	(0.59 - 0.74)	<.0001*
Dependent score	0.86	(0.75 - 0.97)	0.0161*
Peer conflict/social withdrawal score	0.66	(0.49 - 0.89)	0.0070*
-	-	-	-
**C3**	-	-	-
Race	-	-	-
Hispanic vs. White, non-Hispanic	0.93	(0.59 - 1.47)	0.6549
Black vs. White, non-Hispanic	0.71	(0.44 - 1.13)	0.1776
Mother’s education level (year)	1.05	(0.97 - 1.14)	0.2291
Mother smoked during 12 months before the birth of the child (yes)	0.68	(0.44 - 1.03)	0.0709
Breastfeed (yes)	0.94	(0.65 - 1.38)	0.7639
Headstrong score	0.69	(0.60 - 0.78)	<.0001*
Dependent score	0.98	(0.84 - 1.14)	0.7887
Peer conflict/social withdrawal score	1.08	(0.80 - 1.47)	0.6014
-	-	-	-
**C5**	-	-	-
Race	-	-	-
Hispanic vs. White, non-Hispanic	1.03	(0.68 - 1.56)	0.1996
Black vs. White, non-Hispanic	1.77	(1.21 - 2.56)	0.0024*
Mother’s education level (year)	1.04	(0.97 - 1.11)	0.3175
Mother smoked during 12 months before the birth of the child (yes)	0.76	(0.53 - 1.07)	0.1138
Breastfeed (yes)	1.10	(0.80 - 1.52)	0.5560
Headstrong score	1.26	(1.12 - 1.42)	<.0001*
Dependent score	1.12	(0.98 - 1.28)	0.0886
Peer conflict/social withdrawal score	1.52	(1.23 - 1.87)	<.0001*
-	-	-	-
**C6**	-	-	-
Race	-	-	-
Hispanic vs. White, non-Hispanic	1.01	(0.56 - 1.82)	0.2242
Black vs. White, non-Hispanic	2.04	(1.23 - 3.38)	0.0048*
Mother’s education level (year)	0.91	(0.83 - 1.00)	0.0595
Mother smoked during 12 months before the birth of the child (yes)	1.34	(0.86 - 2.10)	0.2022
Breastfeed (yes)	1.07	(0.69 - 1.65)	0.7763
Headstrong score	1.96	(1.63 - 2.37)	<.0001*
Dependent score	1.48	(1.22 - 1.78)	<.0001*
Peer conflict/social withdrawal score	1.81	(1.41 - 2.32)	<.0001*

Table TS5. Logistic regression stepwise selected predictors using C5 as the reference group.

-	Odds Ratio	95% CI	p-value
**C1**	-	-	-
Race	-	-	-
Hispanic vs. White, non-Hispanic	0.60	(0.32 - 1.12)	0.8532
Black vs. White, non-Hispanic	0.32	(0.18 - 0.59)	0.0037*
Mother’s education level (year)	1.06	(0.95 - 1.18)	0.2970
Mother smoked during 12 months before the birth of the child (yes)	0.80	(0.45 - 1.45)	0.4661
Breastfeed (yes)	1.70	(1.02 - 2.84)	0.0418*

-	Odds Ratio	95% CI	p-value
Headstrong score	0.28	(0.22 - 0.34)	<.0001*
Dependent score	0.61	(0.49 - 0.77)	<.0001*
Peer conflict/social withdrawal score	0.28	(0.14 - 0.56)	0.0003*
-	-	-	-
**C2**	-	-	-
Race	-	-	-
Hispanic vs. White, non-Hispanic	0.64	(0.40 - 1.03)	0.7588
Black vs. White, non-Hispanic	0.47	(0.31 - 0.72)	0.0113*
Mother’s education level (year)	1.08	(0.99 - 1.17)	0.0709
Mother smoked during 12 months before the birth of the child (yes)	0.96	(0.64 - 1.45)	0.8619
Breastfeed (yes)	0.96	(0.67 - 1.38)	0.8257
Headstrong score	0.52	(0.46 - 0.60)	<.0001*
Dependent score	0.76	(0.66 - 0.89)	0.0004*
Peer conflict/social withdrawal score	0.44	(0.32 - 0.59)	<.0001*
-	-	-	-
**C3**	-	-	-
Race	-	-	-
Hispanic vs. White, non-Hispanic	0.90	(0.53 - 1.53)	0.1646
Black vs. White, non-Hispanic	0.40	(0.24 - 0.67)	0.0005*
Mother’s education level (year)	1.01	(0.93 - 1.11)	0.7555
Mother smoked during 12 months before the birth of the child (yes)	0.90	(0.56 - 1.44)	0.6486
Breastfeed (yes)	0.86	(0.56 - 1.31)	0.4746
Headstrong score	0.54	(0.47 - 0.63)	<.0001*
Dependent score	0.87	(0.74 - 1.04)	0.1245
Peer conflict/social withdrawal score	0.72	(0.53 - 0.97)	0.0322*
-	-	-	-
**C4**	-	-	-
Race	-	-	-
Hispanic vs. White, non-Hispanic	0.97	(0.64 - 1.47)	0.1996
Black vs. White, non-Hispanic	0.57	(0.39 - 0.82)	0.0024*
Mother’s education level (year)	0.97	(0.90 - 1.04)	0.3175
Mother smoked during 12 months before the birth of the child (yes)	1.32	(0.94 - 1.87)	0.1138
Breastfeed (yes)	0.91	(0.66 - 1.25)	0.5560
Headstrong score	0.79	(0.71 - 0.89)	<.0001*
Dependent score	0.89	(0.78 - 1.02)	0.0886
Peer conflict/social withdrawal score	0.66	(0.54 - 0.81)	<.0001*
-	-	-	-
**C6**	-	-	-
Race	-	-	-
Hispanic vs. White, non-Hispanic	0.98	(0.54 - 1.81)	0.7610
Black vs. White, non-Hispanic	1.15	(0.70 - 1.91)	0.5406
Mother’s education level (year)	0.88	(0.80 - 0.97)	0.0105*
Mother smoked during 12 months before the birth of the child (yes)	1.77	(1.12 - 2.81)	0.0154*
Breastfeed (yes)	0.97	(0.62 - 1.51)	0.8847
Headstrong score	1.56	(1.28 - 1.89)	<.0001*
Dependent score	1.32	(1.09 - 1.60)	0.0048*
Peer conflict/social withdrawal score	1.19	(0.94 - 1.51)	0.1442

Table S6. Logistic regression stepwise selected predictors using C6 as the reference group.

-	Odds Ratio	95% CI	p-value
**C1**	-	-	-
Race	-	-	-
Hispanic vs. White, non-Hispanic	0.61	(0.28 - 1.31)	0.6985
Black vs. White, non-Hispanic	0.28	(0.14 - 0.57)	0.0036*

-	Odds Ratio	95% CI	p-value
Mother’s education level (year)	1.20	(1.06 - 1.36)	0.0048*
Mother smoked during 12 months before the birth of the child (yes)	0.45	(0.23 - 0.88)	0.0192*
Breastfeed (yes)	1.76	(0.96 - 3.22)	0.0659
Headstrong score	0.18	(0.14 - 0.23)	<.0001*
Dependent score	0.47	(0.36 - 0.61)	<.0001*
Peer conflict/social withdrawal score	0.23	(0.11 - 0.48)	<.0001*
-	-	-	-
**C2**	-	-	-
Race	-	-	-
Hispanic vs. White, non-Hispanic	0.65	(0.34 - 1.24)	0.9564
Black vs. White, non-Hispanic	0.41	(0.23 - 0.71)	0.0138*
Mother’s education level (year)	1.22	(1.10 - 1.35)	0.0002*
Mother smoked during 12 months before the birth of the child (yes)	0.54	(0.33 - 0.90)	0.0187*
Breastfeed (yes)	0.99	(0.61 - 1.61)	0.9731
Headstrong score	0.34	(0.27 - 0.41)	<.0001*
Dependent score	0.58	(0.47 - 0.71)	<.0001*
Peer conflict/social withdrawal score	0.37	(0.26 - 0.52)	<.0001*
-	-	-	-
**C3**	-	-	-
Race	-	-	-
Hispanic vs. White, non-Hispanic	0.92	(0.46 - 1.82)	0.1782
Black vs. White, non-Hispanic	0.35	(0.19 - 0.65)	0.0009*
Mother’s education level (year)	1.15	(1.03 - 1.29)	0.0146*
Mother smoked during 12 months before the birth of the child (yes)	0.51	(0.29 - 0.89)	0.0170*
Breastfeed (yes)	0.89	(0.52 - 1.50)	0.6497
Headstrong score	0.35	(0.28 - 0.43)	<.0001*
Dependent score	0.66	(0.53 - 0.83)	0.0003*
Peer conflict/social withdrawal score	0.60	(0.43 - 0.84)	0.0032*
-	-	-	-
**C4**	-	-	-
Race	-	-	-
Hispanic vs. White, non-Hispanic	0.99	(0.55 - 1.78)	0.2242
Black vs. White, non-Hispanic	0.49	(0.30 - 0.82)	0.0048*
Mother’s education level (year)	1.09	(1.00 - 1.20)	0.0595
Mother smoked during 12 months before the birth of the child (yes)	0.75	(0.48 - 1.17)	0.2022
Breastfeed (yes)	0.94	(0.61 - 1.45)	0.7763
Headstrong score	0.51	(0.42 - 0.62)	<.0001*
Dependent score	0.68	(0.56 - 0.82)	<.0001*
Peer conflict/social withdrawal score	0.55	(0.43 - 0.71)	<.0001*
-	-	-	-
**C5**	-	-	-
Race	-	-	-
Hispanic vs. White, non-Hispanic	1.02	(0.55 - 1.87)	0.7610
Black vs. White, non-Hispanic	0.87	(0.52 - 1.44)	0.5406
Mother’s education level (year)	1.13	(1.03 - 1.25)	0.0105*
Mother smoked during 12 months before the birth of the child (yes)	0.56	(0.36 - 0.90)	0.0154*
Breastfeed (yes)	1.03	(0.66 - 1.61)	0.8847
Headstrong score	0.64	(0.53 - 0.78)	<.0001*
Dependent score	0.76	(0.63 - 0.92)	0.0048*
Peer conflict/social withdrawal score	0.84	(0.66 - 1.06)	0.1442

Table S7. Sensitivity analysis of model fit for 1-7 class quartic group-based trajectory analysis.

Model	Number of Class	BIC	Jeffreys and Kass and Raftery Approximation 2*(BICj-BICi)	Model Comparison (j to i)	Evidence for or Against
1	One	-10555.82	--	--	--
2	Two	-9507.63	2096.38	Model 2 to model 1	Very strong evidence against model 1
3	Three	-9303.62	408.02	Model 3 to model 2	Very strong evidence against model 2
4	Four	-9243.48	120.28	Model 4 to model 3	Very strong evidence against model 3
5	Five	-9221.02	44.92	Model 5 to model 4	Very strong evidence against model 4
6	**Six**	-9218.49	**5.06**	Model 6 to model 5	Strong evidence against model 5
7	Seven	-9220.17	-3.36	Model 7 to model 6	No evidence against model 6
